# Human cellular and humoral immune responses to *Phlebotomus papatasi* salivary gland antigens in endemic areas differing in prevalence of *Leishmania major* infection

**DOI:** 10.1371/journal.pntd.0005905

**Published:** 2017-10-12

**Authors:** Wafa Kammoun-Rebai, Narges Bahi-Jaber, Ikbel Naouar, Amine Toumi, Afif Ben Salah, Hechmi Louzir, Amel Meddeb-Garnaoui

**Affiliations:** 1 Laboratory of Medical Parasitology, Biotechnologies and Biomolecules, Institut Pasteur de Tunis, Tunis, Tunisia; 2 Département de Biologie, Université Tunis El Manar, Tunis, Tunisia; 3 UPSP EGEAL Institut Polytechnique LaSalle Beauvais, Beauvais, France; 4 Laboratory of Transmission, Control and Immunobiology of Infection, Institut Pasteur de Tunis, Tunis, Tunisia; 5 Arabian Gulf University, College of Medicine and Medical Sciences, Manama, Bahrain; 6 Faculté de Médecine de Tunis, Université Tunis El Manar, Tunis, Tunisia; McGill University, CANADA

## Abstract

**Background:**

Sand fly saliva compounds are able to elicit specific immune responses that have a significant role in *Leishmania* parasite establishment and disease outcome. Characterizing anti-saliva immune responses in individuals living in well defined leishmaniasis endemic areas would provide valuable insights regarding their effect on parasite transmission and establishment in humans.

**Methodology/Principal findings:**

We explored the cellular and humoral immune responses to *Phlebotomus* (*P*.) *papatasi* salivary gland extracts (SGE) in individuals living in cutaneous leishmaniasis (CL) old or emerging foci (OF, EF). OF was characterized by a higher infection prevalence as assessed by higher proportions of leishmanin skin test (LST) positive individuals compared to EF. Subjects were further subdivided into healed, asymptomatic or naïve groups. We showed anti-SGE proliferation in less than 30% of the individuals, regardless of the immune status, in both foci. IFN-γ production was higher in OF and only observed in immune individuals from OF and naïve subjects from EF. Although IL-10 was not detected, addition of anti-human IL-10 antibodies revealed an increase in proliferation and IFN-γ production only in individuals from OF. The percentage of seropositive individuals was similar in immune and naïves groups but was significantly higher in OF. No correlation was observed between anti-saliva immune responses and LST response. High anti-SGE-IgG responses were associated with an increased risk of developing ZCL. No differences were observed for anti-SGE humoral or cellular responses among naïve individuals who converted or not their LST response or developed or not ZCL after the transmission season.

**Conclusions/Significance:**

These data suggest that individuals living in an old focus characterized by a frequent exposure to sand fly bites and a high prevalence of infection, develop higher anti-saliva IgG responses and IFN-γ levels and a skew towards a Th2-type cellular response, probably in favor of parasite establishment, compared to those living in an emerging focus.

## Author summary

During murine experimental leishmaniasis sand fly saliva components modulate the host immune response and facilitate infection while pre-exposition to uninfected sand fly bites is associated with a protective cellular response against subsequent infection. Human anti-saliva immune responses are not well defined in leishmaniasis endemic areas. Here, we report an analysis of anti *P*. *papatasi* saliva cellular and humoral responses in individuals residing in endemic foci showing different prevalence rates of *L*. *major* infection. Individuals were further subdivided based on LST response and presence of typical CL scars. We showed higher anti-saliva cellular and humoral responses and a skew towards a Th2 response in the old focus characterized by the highest prevalence of infection. No correlation was observed between LST and anti-saliva cellular or humoral response. We showed that high anti-saliva IgG responses constituted a risk factor for the development of CL. Our findings suggest that the anti-*P*. *papatasi* saliva cellular and humoral response profiles vary with the level of sand fly exposure and the prevalence of infection in CL endemic areas. Such studies in humans from highly endemic areas could contribute to a better understanding of the immune response to sand fly saliva and its role in leishmaniasis outcome.

## Introduction

Leishmaniasis caused by protozoan parasites of the genus *Leishmania* transmitted by phlebotomine sand fly vectors has one of the largest diseases burden among the neglected tropical diseases [1,2]. These infections cause a broad clinical spectrum including cutaneous, mucocutaneous or visceral forms with variable severity, depending on the parasite species and the host immune status [3,4]. Leishmaniasis can also be asymptomatic in humans [4–6]. Disease control is mainly based on surveillance of incident cases and treatment, which is expensive, toxic and often associated with the emergence of drug-resistant strains [7]. In Tunisia, zoonotic cutaneous leishmaniasis (ZCL) due to *Leishmania* (*L*.) *major* is the most frequent clinical form. Thousands of cases are reported every year since its first emergence as an epidemic in central Tunisia in 1982. The disease has spread in many parts of the country, with the emergence of several new foci and constitutes a public health problem [8–10]. *L*. *major* is transmitted by the bite of *Phlebotomus* (*P*.) *papatasi* [11,12]. Sand fly bite is a critical event in *Leishmania* transmission and saliva of this vector is a determining factor in infection. It has been shown that co-inoculation of *Leishmania* parasites with saliva enhances disease progression [13–16]. These exacerbating effects have been attributed to salivary proteins such as maxadilan, a vasodilator in *Lutzomyia (Lu*.*) longipalpis* saliva, and associated with immuno-modulatory activities, including inhibition of IFN-γ ability to activate macrophages to kill the intracellular parasite, up-regulation of Th2 cytokines production, down-regulation of some molecules important in parasite destruction such as nitric oxide and alterations in dendritic cell phenotype and function [15–22]. However, it has also been demonstrated that repeated exposure to uninfected sand fly bites elicits saliva-specific Th1-mediated delayed-type hypersensitivity (DTH) responses that were associated with protection against *Leishmania* infection in mice [15,23,24]. Immunization with individual salivary proteins was shown to induce distinct immune profiles that correlated with resistance or susceptibility to *L*. *major* infection in animal models [25–29]. DTH to sand fly bites was also described in humans [24,30] and more recently in a cutaneous leishmaniasis (CL) endemic area where it was shown to be Th1-mediated [31]. However, effects of sand fly saliva during natural exposure of individuals in leishmaniasis endemic areas, on modulation of parasite-specific immune response and outcome of infection, are not clearly understood. Field studies have demonstrated the presence of anti-saliva antibodies in humans naturally exposed to sand fly bites [32–35]. The use of antibodies against sand fly saliva or against salivary recombinant proteins as potential markers of exposure was suggested in endemic areas of leishmaniasis [34–39]. Antibodies to saliva might also be used as markers for the risk of *Leishmania* infection [34,35,40]. Few studies have assessed human cellular immune responses to sand fly saliva and its effects on parasite establishment. PBMC from human volunteers experimentally exposed to *Lu*. *longipalpis* bites displayed an increased frequency of CD4+ and CD8+ T cells as well as an increase in IFN-γ and IL-10 production upon stimulation with saliva [30]. Analysis of cellular immune responses against *P*. *papatasi* saliva in PBMC from individuals naturally exposed to *L*. *major* infection, showed low proliferation, absence of IFN-γ production but significant IL-10 levels, which could favor establishment of infection [41].

To better understand the impact of host immune response to *P*. *papatasi* saliva on *L*. *major* infection development and to determine potential correlations between anti-saliva immune response and leishmaniasis outcome during natural exposure, we have investigated here the humoral and proliferative responses, as well as cytokine production to *P*. *papatasi* salivary gland extracts, in a large cohort of individuals living in CL old or emerging foci differing in prevalence of *L*. *major* infection, in Tunisia.

## Materials and methods

### Ethics statement

This research was conducted with the approval of the local ethical Committee of the Pasteur Institute of Tunis (protocol number 07–0018). Written informed consent was obtained from all individuals and from parents or legal guardians in case of minors before enrollment.

### Study area and target population

**Fig 1:**
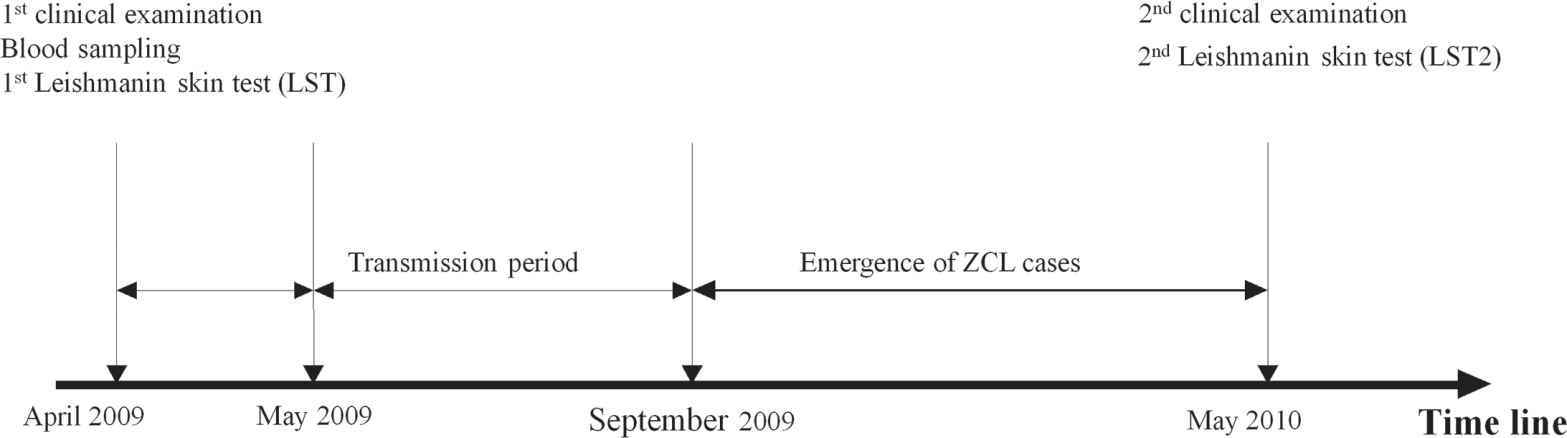
Timeline showing the different steps of the prospective study. 790 participants living in endemic areas of ZCL were followed up over 1 year throughout one season of *L*. *major* transmission. Parameters such as LST and the presence of typical scars were monitored at the beginning of the study and after the transmission season and the triggering of new cases. Peripheral blood samples were obtained from each donor at the beginning of the study.

Our cohort consisted of 790 individuals aged from 6 to 20 years (mean age 12.5+/-3.5) randomly selected from 5 zoonotic foci of CL due to *L*. *major* situated in central Tunisia. During this prospective study, a specific questionnaire to collect informations on past history of ZCL, clinical examination for the presence of typical scars leishmanin skin test (LST) (for detection of exposure to *Leishmania* parasites) and peripheral blood sampling were performed for all individuals, in April, prior to the transmission season of CL, which occur between June and October [9] (**Fig 1**).

The LST was considered positive if the mean of the 2 diameters of induration was five mm or more [42,43]. A clinical follow up of one year throughout one *L*. *major* transmission season and a second LST (LST2) as well as the detection of new CL cases, were carried out. 29 new active CL cases were detected during the subsequent transmission season. CL diagnosis was based on clinical criteria and the demonstration of *Leishmania* parasites in Giemsa-stained dermal smears by microscopy.

All individuals taking part in our study come from a larger cohort of individuals living in the 5 foci of CL that have been recently analyzed during an LST-epidemiological study attempting to estimate the prevalence of *L*. *major* infection based on LST reactivity [9]. Mnara was an old-focus (OF) whereas, Mbarkia, Dhouibet, Msaâdia and Ksour were considered as emerging foci (EF), on the basis of case notification data in the district epidemiological surveillance system. There were no significant differences between the 5 foci regarding demographic and socioeconomic characteristics. Bettaieb et al. demonstrated a significantly higher prevalence of infection in the old focus whereas no differences regarding this parameter were observed between the 4 emerging foci [9]. For our cohort, we further compared the clinical and immunological parameters related to exposure and infection by *Leishmania*, namely the median size of the LST and LST2, the percentage of individuals with positive LST and LST2, the median number of scars and lesions per individual, the percentage of individuals with scars, the percentage of healed (LST+/Scar+), asymptomatic (LST+/Scar-) and naïve (LST-/Scar-) individuals and the number of active ZCL cases, between the four emerging foci, and showed no significant differences (**Table 1**).

**Table 1:** Clinical and immunological parameters in individuals living in various emerging foci of *L*. *major* infection.

	Dhouibet	Ksour	Msaadia	Mbarkia	p-value
LST size reaction (median/IQR)	0/10	0/10	0/6.5	0/9.5	NS (**)
% LST positive individuals	41.5 (66/159)	43.5 (98/225)	39.3 (48/122)	38 (38/100)	NS
LST2 size reaction (median/IQR)	2/12.5	0/11	0/6.5	0/11.5	NS
% LST2 positive individuals	46.7 (57/122)	47 (88/187)	60 (66/110)	49.4 (42/85)	NS
Number of scars/individual (median/IQR)	2/2	2/1	2/1	1/0	NS
% of individuals with scars	20.7 (33/159)	23.1 (52/225)	14.7 (18/122)	13 (13/100)	NS
Number of lesions/ individual (median/IQR)	0/0	0/0	0/0	0/0	NS
% individuals with active CL	6.9 (11/159)	3.5 (8/225)	2.4 (3/122)	5 (5/100)	NS
% LST+/Scar+ (*)	15.7 (25/159)	17.3 (39/225)	13.1 (16/122)	11 (11/100)	NS
% LST+/Scar- (*)	25.7 (41/159)	26.2 (59/225)	26.2% (32/122)	27 (27/100)	NS
% LST-/Scar- (*)	53.4 (85/159)	50.6 (114/225)	59 (72 /122)	60 (60/100)	NS
Number of individuals	159	225	122	100	

(*) 15 LST-/Scar+ individuals from EF foci were not included when LST+/Scar+, LST+/Scar-and LST-/Scar- groups, were considered, which explains that the total number of the 3 combined groups does not correspond to the total number of individuals in EF.

(**) Not significant

However, these parameters were significantly different between the old focus and combined emerging foci, especially for LST size reaction (median/IQR: 12/4 and 0/9, respectively) and percentage of individuals with positive LST (98.3% and 41.2%, respectively) (**Table 2**). Taking these data into account, individuals from the old focus (OF) and those from emerging combined foci (EF) were considered separately.

**Table 2:** Clinical and immunological parameters in individuals living in old (OF) or emerging foci (EF) of *L*. *major* infection.

	OF	EF	p-value
LST size reaction (median/IQR)	12/4	0/9	0.0001
% LST positive individuals	98.3% (181/184)	41.2% (250/606)	<0.0001
LST2 size reaction (median/IQR)	12.5/3	0/11.5	0.0001
% LST2 positive individuals	100% (153/153)	45.8% (231/504)	0.0001
Number of scars/individual (median/IQR)	1/1	2/1	NS (**)
% of individuals with scars	40.2% (74/184)	19.1% (116/606)	<0.0001
Number of lesions/ individual (median/IQR)	0/0	0/0	0.0001
% individuals with active CL	1.09% (2/184)	4.4% (27/606)	0.02
% LST+/Scar+ (*)	40.2% (74/184)	16.6% (101/606)	0.0000
% LST+/Scar- (*)	59.2 (109/184)	26.2 (159/606)	0.0000
% LST-/Scar- (*)	0.54 (1/184)	54.6 (331/606)	0.0000
Number of individuals	184	606	

(*) 15 LST-/Scar+ individuals from EF foci were not included when LST+/Scar+, LST+/Scar-and LST-/Scar- groups, were considered, which explains that the total number of the 3 combined groups does not correspond to the total number of individuals in EF.

(**) Not significant

Human groups were defined based on the following inclusion criteria (i) living in endemic area of *L*. *major* infection, information on past history of ZCL based on a specific questionnaire, positive LST reaction and presence of typical CL scars (LST+Scar+) for healed individuals (ii) living in endemic area of *L*. *major* infection, positive LST reaction and absence of typical CL scars (LST+Scar-) for individuals with a probable asymptomatic infection and (iii) living in endemic area of *L*. *major* infection, negative LST reaction and absence of typical CL scars (LST-Scar-) for naïve individuals.

### Study design

We first analyzed anti-SGE humoral and cellular responses in all individuals from OF (n = 184, n = 82; respectively) and EF (n = 606, n = 312; respectively), regardless of their response to LST or the presence of scars (**Table 3**). In a second step we performed this analysis in LST+/Scar+ (humoral/cellular responses performed in 74/33 individuals), LST+/Scar- (109/49) groups from OF and LST+/Scar+ (101/45), LST+/Scar- (159/79) and LST-/Scar- (331/173) groups from EF. Luminex assay was performed in a subgroup of 58 EF individuals subdivided into 17 LST+/Scar+, 23 LST+/Scar- and 18 LST-/Scar- (**Table 3**).

**Table 3:** Study design.

Anti-SGE IgG responses analysis (N = 790)	OF			EF		
	N = 184			N = 606		
	LST+Scar+	LST+Scar-	LST-Scar-	LST+Scar+	LST+Scar-	LST-Scar-
	N = 74	N = 109	N = 1	N = 101 (*)	N = 159	N = 331
Anti-SGE cellular responses analysis (N = 395)	OF			EF		
	N = 82			N = 312		
	LST+Scar+	LST+Scar-	LST-Scar-	LST+Scar+	LST+Scar-	LST-Scar-
	N = 33	N = 49	N = 0	N = 45 (*)	N = 79	N = 173
Cytokines/chemokines in response to SGE by Luminex (N = 58)	EF					
	N = 58					
	LST+Scar+		LST+Scar-		LST-Scar-	
	N = 17		N = 23		N = 18	

(*) 15 LST-/Scar+ individuals from EF foci were not included when LST+/Scar+, LST+/Scar-and LST-/Scar- groups, were considered, which explains that the total number of the 3 combined groups does not correspond to the total number of individuals in EF.

### Salivary gland extracts preparation

Sand fly salivary glands were kindly provided by E. Zhioua and S. Cherni (Laboratory of Vector Ecology, Pasteur Institute of Tunis). They were dissected out from a colony of the Tunisian *P*. *papatasi* vector of *L*. *major* and stored in groups of 20 pairs in 20μl phosphate buffered saline (PBS). Immediately before use, salivary glands were disrupted by 5 freezing/thawing cycles. After centrifugation, the supernatants were stored at –80°C with 10% glycerol. Just before use, SGE was prepared by dilution in cell culture medium added with gentamycin (Invitrogen).

### Lymphoproliferative responses

Peripheral Blood Mononuclear cells (PBMC) were isolated from blood by density centrifugation through Ficoll-Hypaque (Pharmacia, Uppsala, Sweden). Cells were resuspended in RPMI 1640 culture medium supplemented with 1% Hepes 1M, sodium Pyruvate 1mM, 1X non essential amino acids, 0.1% β mercapto-ethanol 50mM, 10% heat inactivated AB human serum, 2mM L-glutamine, 100 IU/ml penicillin and 100 mg/ml streptomycin. Purified blocking anti-human IL-10 antibody (BD Biosciences, Le Pont de Claix, France) was used in some cell culture conditions. PBMC were cultured at 10^5^ cells/well in triplicates in 96 well plates in a final volume of 200 μL and incubated with SGE (1gland/ml) with or without an anti-IL10 antibody (500ng/ml), at 37°C, in a 5%CO2 humidified atmosphere, for 5 days. Purified Protein Derivative (PPD) (5μg/ml) was used as a positive control. Control experiments were also performed on non-stimulated PBMC. During the last 10 hours of incubation, 0.4μCi/well of 3H-thymidine (Amersham) was added to cultures. The cells were harvested on glass-fiber filters using a multichannel cell harvester (PHD cell harvester, Cambridge technology) and ^3^H-thymidine incorporation was measured by liquid scintillation counter (Rack Beta; LKB wallace). Results were expressed as stimulation index (SI) obtained by dividing the mean counts of triplicates in antigen-stimulated cultures by the mean counts of triplicates in non-stimulated cultures. Lymphoproliferation was considered positive when SI was superior or equal to 2.

### Cytokine detection by ELISA

IL-10 or IFN-γ levels were measured by Enzyme-linked immunosorbent assay (ELISA) in cell culture supernatants collected after 48h or 72h, respectively, centrifuged and stored at -80°C until use. Human IL-10 or IFN-γ ELISA Sets (BD Biosciences) were used according to manufacturer’s instructions. For each cytokine determination, the results were interpolated from a standard curve using recombinant cytokines and expressed in pg/mL.

### Analysis of antibody response against SGE

Specific anti-saliva IgG antibodies were measured by ELISA. The wells (NUNC, Maxisorp Roskilde, Denmark) were coated with SGE (0.5 glands/well) in 0.1 M carbonate-bicarbonate buffer (pH 9.6) overnight at 4°C. After three washes with PBS-0.05% Tween, free binding sites in the plates were blocked for 1 hour at 37°C with PBS containing 0.1% Tween 20 and 0.5% gelatine (PBS-T-G). Human sera were diluted (1:200) in PBS-T-G and incubated for 2 hours at 37°C. After washing, wells were incubated with peroxidase-conjugated anti-human IgG antibodies (Sigma) at a 1:5000 dilution in PBS-T-G, for 1 hour at 37°C. Tetramethylbenzidine (TMB) substrate (Sigma) was added for 10 min at room temperature to visualize antibody-antigen complexes. The absorbance was measured using an automated ELISA reader (Thermo Labsystems Multiskan Ex) at 450 nm to 620 nm wavelengths. All sera were tested in duplicate and the mean value was recorded. Results were expressed as relative OD (ROD) defined as the ratio of sample OD/mean OD of sera from 20 negative controls (+ two standard deviations). ROD superior or equal to 2 was considered positive. Negative sera were obtained from healthy controls living outside Tunisia in sand fly-free regions.

### Cytokines and chemokines measurements by Luminex assay

Seventeen cytokines and chemokines (IFN-γ, IL-1β, IL-2, IL-4, IL-5, IL-6, IL-10, IL-12p70, IL-13, GM-CSF, TNFα, IL-18, MIP- 1α, IL-8, IL-17A, MCP-1, M-CSF) were analysed using the Luminex multiplex bead-based technology, in culture supernatants from cells stimulated for 48h with SGE, according to the manufacturer’s instructions (Affymetrix, eBioscience).

### Statistical analysis

Data analysis was performed with Stata statistical software (StataCorp. 2009. Stata Statistical Software: Release 11. College Station, TX: StataCorp LP.). Results were expressed as medians (interquartile range, IQR as variances). P values less than 0.05 were considered statistically significant. We used Wilcoxon signed-rank or Mann-Whitney test to determine intragroup differences of cytokine median levels (i.e., differences between stimulated and non-stimulated cultures). Kruskal-Wallis rank test was used for intergroup analysis on normalized data after deducting the non-stimulated value. Proportions for categorical variables were compared using chi-square test. Correlations were estimated using the Spearman rank (rs) correlation coefficient.

Logistic regression was applied to identify risk factors of ZCL cases. Univariate analysis for the following variables was used: age, gender, LST, presence of scars, foci, proliferative responses to (SI <2 and > = 2), IFN-γ responses to SGE, and anti-SGE IgG responses (ROD <2 and > = 2). Variables in univariate analysis with p values less than 0.25 were included in binary multivariate logistic regression. The final model was obtained by backward selection using a significance level of 5%.

## Results

### Cell proliferation to *P*. *papatasi* SGE in individuals living in old versus emerging foci for ZCL

**Fig 2:**
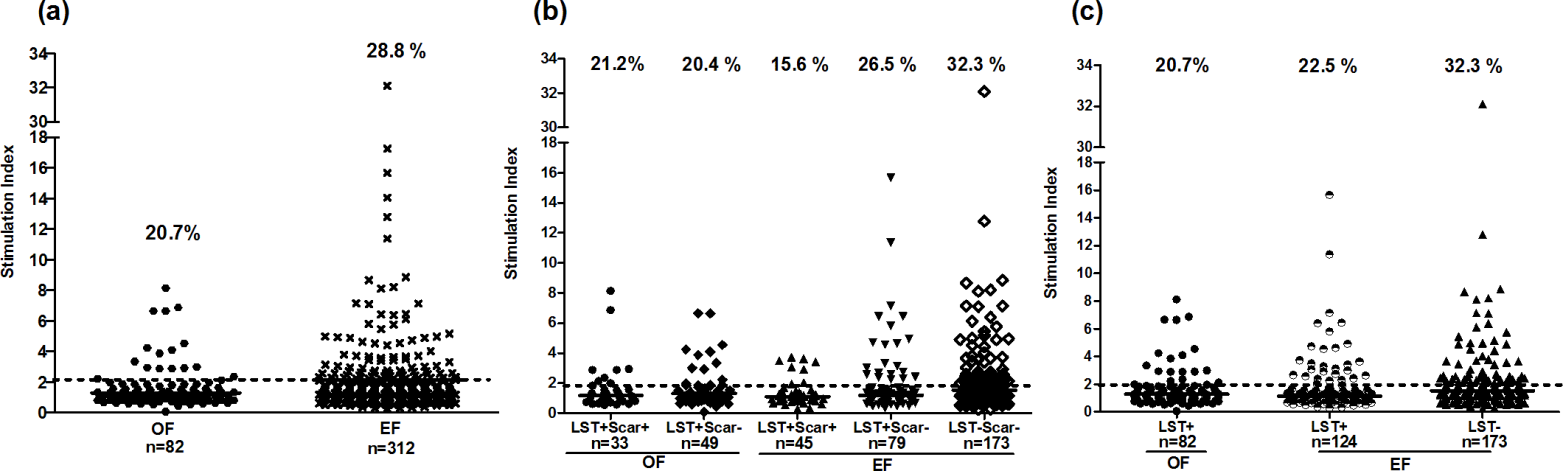
Proliferative responses to *P*. *papatasi* salivary glands extracts in individuals living in old or emerging foci of *L*. *major* infection. PBMC were stimulated for 5 days with *P*. *papatasi* salivary gland extracts (1gland/ml). Proliferation was analyzed in individuals from donors residing in an old (OF) or an emerging focus (EF) (a), LST+/Scar+ (healed), LST+/Scar- (asymptomatics) and LST-/Scar- (naïve) groups (b) and LST+, LST- groups (c), in each focus. Results were expressed as stimulation index (SI) obtained by dividing the mean counts of triplicates in antigen-stimulated cultures by the mean counts of triplicates in non-stimulated cultures. The proliferative response was considered positive when SI was superior or equal to 2. Statistical significance was assigned to a value of p<0,05. Horizontal lines represent median SI values and dotted lines represent cut-off level. The percentages of positive responders are presented for groups and foci.

Proliferative responses to SGE were evaluated in 82 individuals living in OF and 312 individuals living in EF. Positive proliferative responses (IS≥2) were observed in 20.7% (17/82) and 28.8% (90/312) of individuals with median SI/IQR of 3.3/2.7 and 3.1/2.5 in OF and EF, respectively (**Fig 2A**). No significant differences were observed between both foci. In OF, where only asymptomatic (n = 49) and healed individuals (n = 33) were present, the percentage of positive SGE responders and median SI were similar in both groups (20.4% (10/49); 3.9/2.1 and 21.2% (7/33); 2.8/4.5, respectively) (**Fig 2B**). In EF, where naïves (n = 173), asymptomatics (n = 79) and healed (n = 45) were present, higher percentage of SGE positive responders was observed in naïve group (32.3%: 56/173) compared to asymptomatic (26.5%: 21/79) and healed (15.6%: 7/45) groups. Among the immune subjects, the proportion of positive responders was higher in asymptomatic individuals. However, differences were not statistically significant and median SI was similar in the three groups (3/2.5 in naïves; 3.2/3.6 in asymptomatics and 3.4/0.7 in healed individuals) (**Fig 2B**). Furthermore, no significant correlation was found between proliferative responses to SGE and LST in both foci. We further analyzed proliferation results, in EF, by grouping the immune individuals into a single group, based only on the LST response (n = 124 for LST+ individuals). We showed again higher but not significant percentage of SGE positive responders in naïve individuals (32.3%) compared to immune subjects (22.5%) (**Fig 2C**).

### Cytokine responses to *P*. *papatasi* SGE in individuals living in old versus emerging foci for ZCL

**Fig 3:**
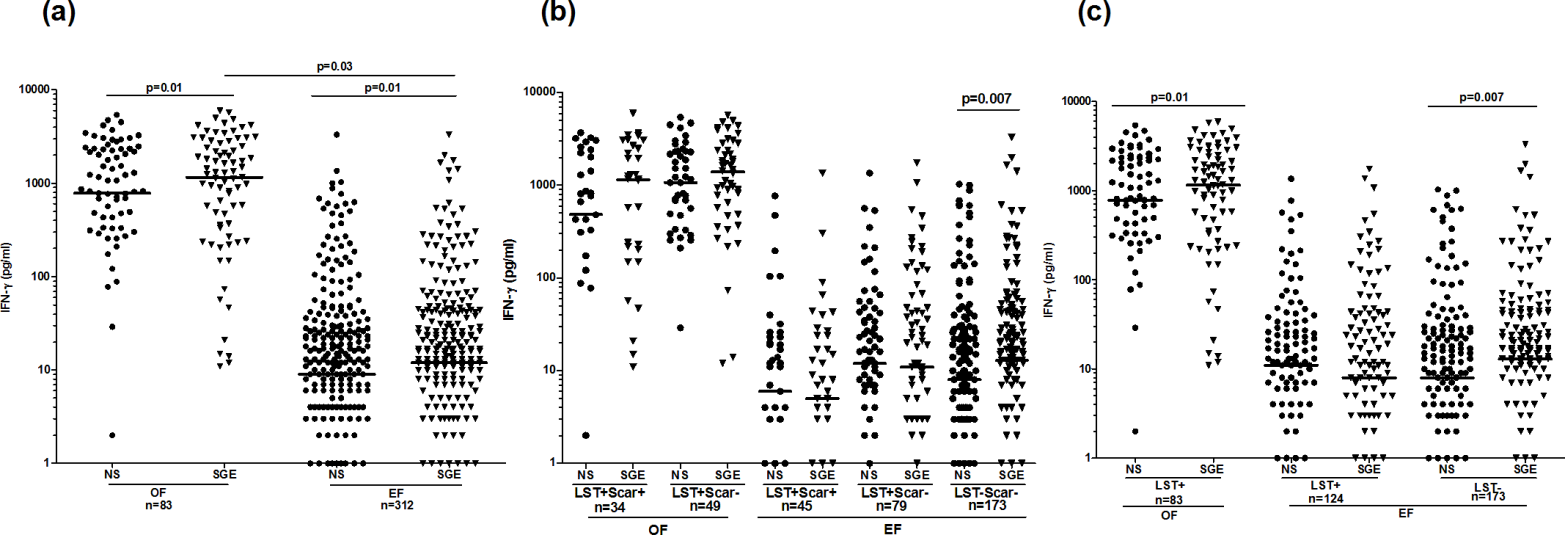
IFN-γ levels induced by *P*. *papatasi* salivary glands extracts stimulation in individuals living in old or emerging foci of *L*. *major* infection. IFN-γ was quantified by ELISA, in the culture supernatants of PBMC stimulated with *P*. *papatasi* salivary gland extracts (1 gland/ml) during 72h. Analysis of IFN-γ production was performed in individuals from OF and EF (a), LST+/Scar+ (healed), LST+/Scar- (asymptomatics) and LST-/Scar- (naïve) groups (b) and LST+, LST- groups (c), in each focus. Statistical significance was assigned to a value of p<0,05. Statistically significant differences between stimulated and non-stimulated cultures and between groups are showed. Horizontal lines represent median IFN-γ values.

IFN-γ and IL-10 responses to SGE were analyzed by ELISA on the same individuals that were evaluated for proliferative responses to saliva. Significant IFN-γ levels were observed in response to SGE stimulation when compared to non-stimulated cultures, in OF (median/IQR: 1136/2307; 775/2016 pg/ml, respectively) and EF (12/39; 9/26 pg/ml) (p = 0.01), (**Fig 3A**). These levels were significantly higher in OF compared to EF (p = 0.03). IFN-γ levels were only significant in individuals with positive proliferation, in both foci (OF (stimulated, non-stimulated cultures): 1744/2363, 665/1308 pg/ml, p = 0.0009; EF: 20/84.7, 9/28 pg/ml, p = 0.0000). A positive correlation was found between SGE proliferative response and IFN-γ production in both OF (r = 0.22; p = 0.047) and EF (r = 0.26; p<0.0001). Interestingly, analysis of IFN-γ responses to SGE according to LST response and presence of scars revealed significant IFN-γ levels only in naïves individuals from EF (13/41, 8/25 pg/ml, in stimulated and non-stimulated cultures, p = 0.007) (**Fig 3B**). We did not detect IFN-γ response in asymptomatics and healed individuals in both foci. However, when we analyzed again IFN-γ response in groups subdivided only on the basis of the LST response, we observed a significant IFN-γ production in LST+ (1152/2307 and 778/2017; p = 0.01) group, only in OF (**Fig 3C**). The difference between this result and the one observed in groups subdivided according to LST and scars could be explained by a lower number of individuals in the latter groups. No correlation was found between anti-SGE IFN-γ response and LST in both EF and OF.

*In vitro* SGE stimulation did not cause a significant change in IL-10 production, when compared to non-stimulated cultures in OF and EF. Furthermore, no significant IL-10 levels were observed in response to SGE in healed, asymptomatic and naïves individuals.

In order to extend the analysis of cytokine response to *P*. *papatasi* saliva, additional cytokines and chemokines (IFN-γ, IL-10, IL-1β, IL-2, IL-4, IL-5, IL-6, IL-12p70, IL-13, GM-CSF, TNFα, IL-18, MIP-1α, IL-8, IL-17A, MCP-1, M-CSF) were evaluated, using Luminex, in culture supernatants from cells issued from 17 healed, 23 asymptomatic and 18 naïve individuals (randomly selected among the EF subjects) and stimulated for 48h with SGE. None of these cytokines or chemokines was detected at significant levels, in response to SGE stimulation. The detection of significant levels of IFN-γ by ELISA but not by Luminex, could be explained by the lower number of individuals analyzed using the Luminex assay.

### Cellular responses to SGE in the presence of an anti-IL10 antibody

**Fig 4:**
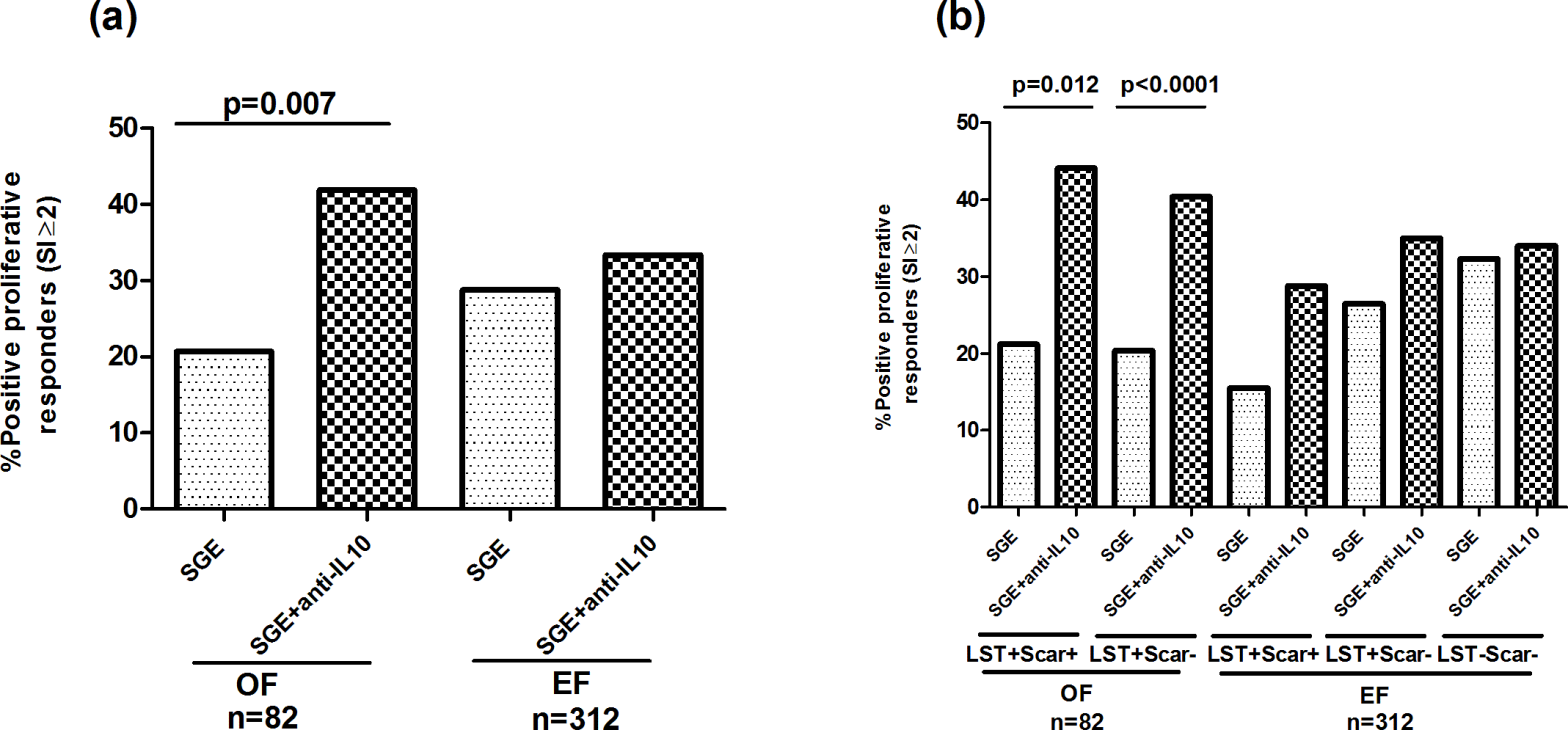
Proliferative responses to *P*. *papatasi* salivary glands extracts in the presence of an anti-IL-10 antibody, in individuals living in old or emerging foci of *L*. *major* infection. PBMC were stimulated with *P*. *papatasi* salivary glands extracts (1gland/ml) with or without an anti-IL10 antibody (500ng/ml), for 5 days. Proliferation was analyzed in individuals from donors residing in an old (OF) or an emerging focus (EF) (a) and LST+/Scar+ (healed), LST+/Scar- (asymptomatics) and LST-/Scar- (naïve) groups (b), in each focus. Results were expressed as percentage of positive proliferative responders. The proliferative response was considered positive when SI was superior or equal to 2. Statistical significance was assigned to a value of p<0,05. Statistically significant differences between groups are showed.

Although we have demonstrated the presence of a cellular response against *P*. *papatasi* saliva in individuals living in endemic areas of CL, both in terms of proliferation and IFN-γ production, this response was generally low. It was shown that *P*. *papatasi* saliva was able to induce the activation of IL-10 producing T cells in naturally exposed individuals [41]. Although we did not detect any significant IL-10 production in response to SGE, we further analyzed proliferation and IFN-γ production to SGE in the presence of an anti-human IL-10 antibody, in order to determine whether the observed SGE-specific cellular responses are down-regulated. We observed that addition of anti-IL-10 antibody significantly increased cellular proliferation only in the old focus (percentage of positive responders: 41.9% and 20.7%, respectively with or without anti-IL-10 antibody, p = 0.007) (**Fig 4A**). A significant increase was also observed in the percentage of positive responders in asymptomatic and healed groups from OF (40.4 and 20.4%, p<0.0001 in asymptomatic group; 44.1 and 21.2%, p = 0.012, in healed group, respectively with or without anti-IL-10 antibody (**Fig 4B**). Anti-IL-10 antibody had no significant effect on the cellular response against SGE in individuals from the emerging focus. Median SI values were not affected by anti-IL-10 antibody addition, in all groups and foci.

**Fig 5:**
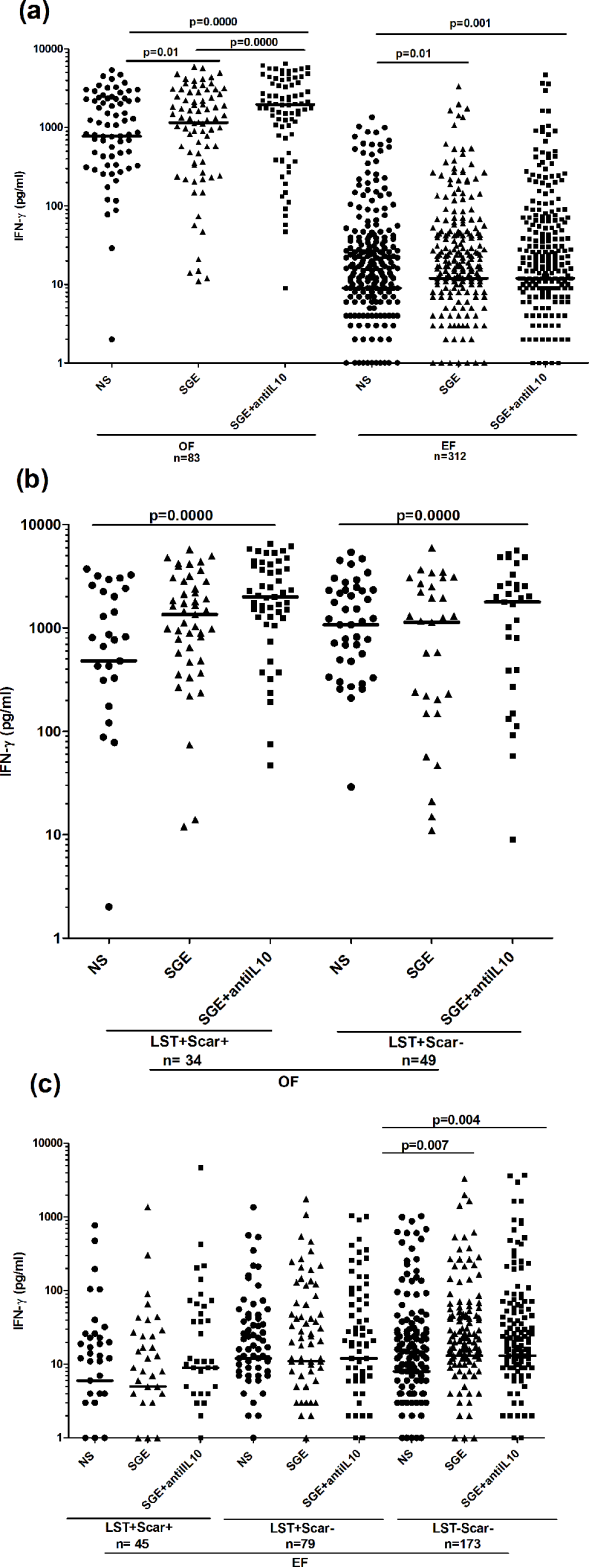
IFN-γ levels induced by *P*. *papatasi* salivary glands extracts stimulation in the presence of an anti-IL-10 antibody, in individuals living in old or emerging foci of *L*. *major* infection. IFN-γ was quantified by ELISA, in the culture supernatants of PBMC stimulated with *P*. *papatasi* salivary gland extracts (1 gland/ml), with or without an anti-IL10 antibody (500ng/ml), during 72h. Analysis of IFN-γ production was performed in individuals from OF and EF (a) and LST+/Scar+ (healed), LST+/Scar- (asymptomatics) and LST-/Scar- (naïve) groups in OF (b) and EF (c). Statistical significance was assigned to a value of p<0.05. Statistically significant differences between stimulated and non-stimulated cultures and between groups are showed. Horizontal lines represent median IFN-γ values.

IFN-γ production was significantly increased when anti-IL-10 antibody was added to cultures, in individuals from OF (712/1491 and 30/784, p = 0.0000, respectively with or without anti-IL-10 antibody) but not in those from EF (**Fig 5A**). Interestingly, addition of anti-IL-10 antibody allowed to detect significant levels of IFN-γ in asymptomatic (2004/2786, 1071/1995; in stimulated and non-stimulated cultures, p = 0.0000) and healed (1740/2483, 455/1943; p = 0.0000) groups from OF (**Fig 5B**). This cytokine was not detected in these groups, in the absence of anti-IL-10 antibody. Anti-IL-10 antibody did not affect IFN-γ production in groups from EF (**Fig 5C**).

### IgG responses to *P*. *papatasi* salivary gland extract in individuals living in old versus emerging foci for ZCL

**Fig 6:**
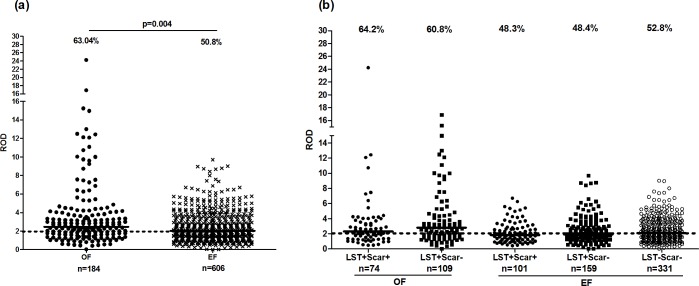
IgG response against *P*. *papatasi* salivary glands extracts in individuals living in old or emerging foci of *L*. *major* infection. Analysis of IgG response was performed in individuals from OF and EF (a) and LST+/Scar+ (healed), LST+/Scar- (asymptomatics) and LST-/Scar- (naïve) groups (b), in each focus. IgG antibodies were measured by ELISA. Results were expressed as relative OD (ROD) defined as the ratio of sample OD/mean OD of sera from negative controls. ROD superior or equal to 2 was considered positive. Negative sera were obtained from healthy controls living outside Tunisia in sand fly-free regions. Statistical significance was assigned to a value of p<0,05. Statistically significant differences between groups are showed for positive responders. Horizontal lines represent median ROD values and dotted lines represent cut-off level. The percentages of positive responders are presented for groups and foci.

We measured the reactivity of SGE, using plasma samples from 184 and 606 individuals living in OF and EF foci, respectively. We showed a significantly higher proportion of seropositive individuals in the old focus (63.04%, 116/184) compared to the emerging one (50.8%, 308/606), (p = 0.004), (**Fig 6A**). Median ROD/IQR was also significantly higher in OF (2.46/2.3), in comparison with EF (2.02/2) (p = 0.0006). However, median ROD of seropositive individuals was similar in both foci (OF: 3.2/2.5; EF: 3.2/1.8). Furthermore, no significant differences were observed in terms of proportion of seropositive individuals or median ROD, between asymptomatic (n = 109) (64.2%; 3.31/3.4) and healed (n = 74) (60.8%; 3.16/1.9) groups from OF or asymptomatic (n = 159) (48.4%; 3.04/1.9, healed (n = 101) (48.3%; 2.8/1.9 and naïve (n = 331) (52.8%; 3.5/1.8) groups from EF (**Fig 6B**). No correlation was found between anti-saliva IgG response and LST in both foci.

In addition, we showed that the percentage of individuals with positive proliferation against SGE (SI+) was significantly higher among seropositive individuals (ROD+) compared to seronegative individuals (ROD-) in both foci (%SI+ in ROD+ and ROD-: 25% and 5.8% in OF; 39.5% and 16.5% in EF). Significant levels of IFN-γ were only observed in seropositive individuals in both foci (median IFN-γ/IQR in stimulated and non-stimulated cultures: 1038/2019, 689/1346; p = 0.001 in OF and 13/47, 9/28; p = 0.004 in EF). Significant correlations were found between SGE specific IgG responses and proliferative responses in both foci (OF: r = 0.38, p = 0.0004; EF: r = 0.3116, p <0.0001), and between IgG responses and IFN-γ responses only in EF (r = 0.16, p = 0.005). However, these correlations were not detected when asymptomatic, healed and naïve groups were considered.

### Evaluation of the effects of specific-SGE cellular and humoral responses on LST conversion and on ZCL development

During the follow-up of one year throughout a *L*. *major* transmission season, a second LST (LST2) evaluation was carried out in individuals from both foci. To estimate the impact of the anti-saliva cellular response (proliferation and IFN-γ) on the conversion of the LST response, we compared the proportion of EF naïve individuals who converted their LST response without developing ZCL after a transmission season (LST-/LST2+/ZCL-) between naïve individuals with positive proliferation to saliva (n = 56) and those with no proliferation (n = 117). Despite a higher percentage of LST-/LST2+/ZCL- individuals in the first group (14.2%) compared to the second one (9.4%), no statistically significant differences were observed. IFN-γ was not detected in naïve individuals, whether or not they have converted their LST response, after a transmission season. Anti-IL-10 had no effect on IFN-γ response in these groups.

We also compared the percentage of EF naïve individuals who developed ZCL after a transmission season (LST-/ZCL+), between naïve individuals with positive proliferation to saliva and those with no proliferation, cited above. Out of 56 naïve individuals with positive saliva proliferation, no individual developed ZCL. All ZCL cases (n = 6) were observed among naïve individuals with no proliferation against SGE (5.12%, 6/117). However, no significant differences were detected between the two groups. Furthermore, IFN-γ was not detected in individuals who have developed ZCL. However, significant IFN-γ levels were observed in the group of individuals who remained naïves (n = 167) (14/42, 8/26, in stimulated compared to non-stimulated cultures, p = 0.01). The addition of anti-IL-10 had no effect on the production of IFN-γ in naïve individuals whether they develop ZCL or not.

No significant differences were observed for percentage of LST-/LST2+/ZCL- individuals between naïve SGE seropositive (10.8%: 19/175) or seronegative groups (8.9%: 14/156). Regarding the effects of anti-SGE humoral responses on the development of ZCL, we did not detect significant differences in the percentage of LST-/ZCL+ individuals between naïve SGE seropositive (2.3%: 4/175) or seronegative groups (1.3%: 2/156).

### Multivariate analysis of risk factors for *L*. *major* infection in individuals exposed to *P*. *papatasi*

We undertook a multivariate analysis using logistic regression to identify risk factors associated with ZCL. During the one-year follow-up, 29 ZCL new cases were detected among individuals from both foci. The anti-SGE antibody response was identified as a risk factor for the development of ZCL. We showed that individuals with mROD≥4 had a risk factor of 2.65 (p = 0.023; 95% confidence interval, 1.14−6.14) to develop ZCL.

## Discussion

Here, we report an analysis of cellular and humoral responses developed against salivary gland extracts from *P*. *papatasi*, in a large cohort of 790 individuals living in areas characterized by hyperendemicity for ZCL infection caused by *L*. *major*. A close spatial association has been reported between the abundance of *P*. *papatasi* and the incidence of ZCL in central and southwestern Tunisia [12]. Our study was conducted in old and emerging foci, in central Tunisia. The determinants of *L*. *major* infection were evaluated for both foci in a recent large LST epidemiologic study, showing a significantly higher prevalence of infection in the old focus as assessed by higher proportions of LST positive individuals compared to the emerging focus [9]. In both foci, individuals were subdivided according to their LST response and the presence of typical ZCL scars into healed, asymptomatic and naïve individuals. Almost all participants had a positive LST, with either healed lesions or asymptomatic infection, in the old focus, whereas more than half of the individuals were naïve in the emerging focus. The LST reaction reflects a CD4+ Th1 cell-mediated immune response [44] and is a useful indicator of immune status in studies evaluating *Leishmania* vaccine candidates [45–47] and an important tool in epidemiological studies to assess exposure to *Leishmania* and the prevalence of infection [9,48–50].

We showed positive proliferative responses against *P*. *papatasi* saliva in less than 30% of individuals with relatively low proliferative indexes and low but significant IFN-γ levels, regardless of the immune status, in both foci. IFN-γ production, which was only observed in individuals with positive proliferation, was significantly higher in the old focus. According to the immune status, anti-saliva proliferation was also similar in healed, asymptomatic and naïve groups from both foci. However, IFN-γ was detected in LST positive individuals (asymptomatic and healed) in the old but not in the emerging focus. We did not observe a correlation between LST response and anti-saliva cellular responses both in terms of proliferation or IFN-γ responses. The observation of higher IFN-γ levels in the old focus suggest a more frequent exposure to sand fly bites in this focus. It has been observed that size of LST reaction, reflecting the time of exposure to the parasite and the intensity of the immune response, increased with lenght of residence in endemic areas [9,51]. In our cohort, the size of LST reaction was significantly higher in individuals from the old focus compared to those from the emerging one, suggesting longer exposure times to sand fly bites and probably higher infection rate of vectors leading to the development of an immunity against infection in almost all individuals living in the old focus. Within the emerging focus, where infecting bites are probably fewer than in the old focus, we showed a significant IFN-γ production only in naïve individuals, suggesting that the anti-saliva cellular immune responses vary depending on whether the host is exposed to saliva in the presence or absence of parasites. Host-parasite interactions within the insect vector before transmission could alter the vector salivary protein profile that may consequently influence the immune response of the human host [52]. A role of the parasite in diminishing host antibody production against a salivary protein was suggested in a study showing a decrease in the immunogenicity of this protein in infected donors compared to donors exposed to sand fly bites but not infected [53].

Few studies have described human cellular responses against sand fly saliva in leishmaniasis endemic areas. A DTH-response (a manifestation of cell-mediated immunity) to *P*. *papatasi*, *L*. *longipalpis* or *P*. *duboscqi* bites, was previously reported in individuals wether after experimental or natural exposure [24,30,31]. An increase in the frequency of T CD4+ and T CD8+ cells, with IFN-γ and IL-10 production were described in PBMC from human volunteers experimentally exposed to *L*. *longipalpis* bites, stimulated with saliva [30]. Similarly to our results, a specific proliferative response to *P*. *papatasi* saliva was showed in 30% of a small group of exposed donors in a CL endemic area and no correlation was observed between these responses and proliferation to soluble *Leishmania* antigens [41]. However, absence of IFN-γ and IL-10 production by T CD8+ cells, in favor a Th2-type cellular response, was observed in this work. Authors showed the ability of activated anti-saliva CD4+ T cells to produce IFN-γ after being separated from CD8+ T cells [41]. IFN-γ, IL-10, IL-5, IL-12 and IL-13 were produced in response to *P*. *duboscqi* saliva stimulation of PBMC from individuals living in a CL endemic area [31]. More recently, a mixed T cell response with a predominance of IL-10 evidenced by elevated IL-10/IFN-γ and IL-10/IL-13 ratios was reported in individuals exposed to *L*. *intermedia* [54]. The predominance of anti-saliva IL-10 response in individuals living in endemic areas led the authors to suggest that exposure to sand fly bites may facilitate *Leishmania* infection [41,54]. It was suggested that IL-10 production could be attributed to the presence of adenosine, a component of *P*. *papatasi* saliva, that have been reported as being able to inhibit NO synthesis and to induce IL-10 production in murine macrophages [55–57]. We did not detect IL-10 production in response to *P*. *papatasi* saliva in our study. Different culture conditions or differences in the sample size of the study groups or studied foci, may explain these different results. Furthermore, variability in the salivary repertoire of various sand fly species or populations that can induce different immune response pattern, may also explain these discrepancies [58–60]. Indeed, salivary molecules associated with immunomodulatory activities, such as adenosine, which is, present in *P*. *papatasi* saliva but absent from *Lu*. *longipalpis* and maxadilan which is only present in *Lu*. *Longipalpis*, has been identified [61]. However, differences in immune responses elicited by salivary proteins from the same sand fly species could be attributed to genetic differences among hosts.

Despite the absence of IL-10 production in the peripheral blood in our study, it cannot be excluded that this cytokine is produced locally, at the site of infection or is present in peripheral blood at undetectable levels. Given the globally low anti-*P*. *papatasi* saliva cellular responses observed in our work and taking into account the published data about this sand fly saliva ability to induce IL-10 in humans, we further analyzed cellular responses using an anti-IL-10 antibody. We showed that addition of anti-IL-10 antibody induced a significant increase in both the percentage of individuals with positive anti-saliva T cell proliferation and IFN-γ production, in asymptomatic and healed groups, in the old focus. This result suggest that, in the old focus, saliva-specific T cell responses could be partially suppressed by IL-10 [62], even though not detected and emphasizes once again the difference between the anti-saliva immune response profiles, depending on the intensity of vector exposure. In endemic areas, individuals are mostly exposed to the bites of uninfected sand flies but are not protected against leishmaniasis. Exposure of mice to short- or long-term *P*. *duboscqi* bites followed by infection either immediately or with a delay, showed protection only in short-term exposed mice, suggesting that chronic exposure to salivary antigens may result in a change in the immune response of the host towards a Th2 profile [63]. Addition of the anti-IL-10 antibody had no effect on cellular responses in individuals living in the emerging focus, suggesting that the absence of IFN-γ observed in the immune group would not be due to its inhibition by immunosuppressive cytokines such as IL-10.

We showed a significantly higher proportion of individuals with positive IgG responses to saliva in the old focus compared to the emerging one, which reveal once again differences in sand fly exposure between both foci. We did not show any change in the anti-saliva humoral response, depending on the LST response. No correlation was observed between anti-saliva cellular or humoral responses and LST, or between anti-saliva cellular and humoral responses, in both foci. The absence of correlation between proliferative and humoral response to *P*. *papatasi* saliva, was reported [41]. Similar anti-*L*. *whitmani* IgG responses were also observed in LST positive and LST negative individuals in a recent study conducted in a CL endemic area with high transmission of *L*. *braziliensis* [64]. However, unlike our findings, LST positivity was correlated to higher levels of *P*. *duboscqi* or *L*. *longipalpis* saliva specific IgG antibodies, in individuals living in CL or VL endemic areas, respectively [32,49]. Differences in salivary proteins from various sand fly species could explain these discrepancies [58–60,65].

Development of IgG antibodies against several sand fly species saliva, and the use of this response as an epidemiological tool for estimating exposure to various sandflies species, has already been reported in leishmaniasis endemic areas [33–36,40,53,54,64,66]. We evaluated the impact of the anti-saliva immune response on the conversion of the LST response and on the development of ZCL in naïve individuals of our cohort. No significant differences were observed for the proportion of individuals who converted their LST response without developing ZCL after a transmission season between individuals with positive proliferation or seropositive to saliva and those with no proliferation or seronegative. IFN-γ was not detected in naïve individuals, whether they converted or not their LST response. These results suggest that the development of a cellular or humoral response to *P*. *papatasi* saliva in naïve individuals, have no impact on the acquisition of a DTH to parasite during a subsequent transmission season, in our emerging focus. It has been reported in animal models that pre-exposition to uninfected sand fly bites is associated with the development of a protective cellular response against *Leishmania* infection [15,18,19,23,25,27,67,68]. However, a lack of protection against a challenge infection has also been reported in mice immunized with sand fly saliva [40]. In VL endemic areas, increased anti-saliva IgG levels in individuals who developed a positive DTH to *L*. *chagasi* antigens, suggesting that the induction of a saliva humoral response could promote induction of a cellular response against the parasite [33,66,69].

We also showed that new ZCL cases were observed among individuals with no proliferation to saliva. Furthermore, no IFN-γ was produced in individuals who developed ZCL while a significant production was detected in those who remained naïve. It would be therefore tempting to suggest that the ability to develop a Th1 cell response against sandfly saliva prior to first contact with the parasite may lead to protection against infection. However, the statistical analysis did not reveal any significant difference, probably due to the low number of new ZCL cases detected during our study period. Our results are in line with data from a study showing that lymphocytes from donors experimentally exposed to sand fly bites seems to limit parasite burden in macrophages in *in vitro* autologous cell cutlture system [30]. Finally, a multivariate analysis of ZCL risk factors showed that individuals with mROD≥4 had a risk factor of 2.65 (p = 0.023) to develop ZCL. Others have reported a correlation between the anti-saliva response and the risk of disease. Higher anti-saliva IgG levels were described in patients with active CL lesions in comparison with healthy or LST positive individuals [34,35,40]. More recently, it has been reported that individuals seropositive to saliva had a higher risk of developing CL than seronegative individuals [54].

Data presented in this study suggest that differences in sand fly exposure levels and in prevalence of infection are associated with different immune responses profiles against *P*. *papatasi* saliva. Higher anti-saliva IgG responses and IFN-γ levels and a skew towards a Th2-type cellular response were observed in immune individuals who are more exposed to sand fly bites and submitted to a higher prevalence of infection. We showed that anti-saliva IgG response did not interfere with the development of a DTH response to parasite but was a risk factor for the development of ZCL due to *L*. *major*. Together, these data can contribute to a better understanding of the mechanisms that govern the resistance or susceptibility to infection by *L*. *major* parasites upon transmission by the *P*. *papatasi* sandflies in endemic areas for ZCL.

## Supporting information

S1 STROBE Checklist (DOC)

S1 Table Epidemiological, clinical and immunological parameters for ZCL cases. (DOCX)
