# Supplementary material for: Human cellular and humoral immune responses to Phlebotomus papatasi salivary gland antigens in endemic areas differing in prevalence of Leishmania major infection
Source: PLoS Negl Trop Dis. 2017 Oct 12;11(10):e0005905. doi: 10.1371/journal.pntd.0005905 (PMC5638224; doi:10.1371/journal.pntd.0005905)
Supplement: S1 STROBE Checklist — (DOC) [file pntd.0005905.s001.doc]

**S1. STROBE checklist for cohort studies**

|  | Item No | Recommendation | Page number and relevant text from manuscript |
| --- | --- | --- | --- |
| **Title and abstract** | 1 | (*a*) Indicate the study’s design with a commonly used term in the title or the abstract | Pages 1,2  Addressed in Title and Abstract |
|  |  | (*b*) Provide in the abstract an informative and balanced summary of what was done and what was found | Pages 2,3  Addressed in Abstract |
| Introduction | | |  |
| Background/rationale | 2 | Explain the scientific background and rationale for the investigation being reported | Pages 4,5  Addressed in Introduction part |
| Objectives | 3 | State specific objectives, including any pre-specified hypotheses | Page 5  Addressed in Introduction part |
| Methods | | |  |
| Study design | 4 | Present key elements of study design early in the paper | Pages 6-8  Section “Study area and target population” |
| Setting | 5 | Describe the setting, locations, and relevant dates, including periods of recruitment, exposure, follow-up, and data collection | Page 6  Section “Study area and target population” |
| Participants | 6 | (*a*) Give the eligibility criteria, and the sources and methods of selection of participants. Describe methods of follow-up | Pages 6,7  Section “Study area and target population” |
|  |  | (*b*) For matched studies, give matching criteria and number of exposed and unexposed | N/A |
| Variables | 7 | Clearly define all outcomes, exposures, predictors, potential confounders, and effect modifiers. Give diagnostic criteria, if applicable | Page 6  For diagnostic criteria |
| Data sources/ measurement | 8* | For each variable of interest, give sources of data and details of methods of assessment (measurement). Describe comparability of assessment methods if there is more than one group | Pages 6-11  Addressed in Methods part |
| Bias | 9 | Describe any efforts to address potential sources of bias | N/A |
| Study size | 10 | Explain how the study size was arrived at | N/A |
| Quantitative variables | 11 | Explain how quantitative variables were handled in the analyses. If applicable, describe which groupings were chosen and why | Pages 11,12  Section “Statistical analysis” |
| Statistical methods | 12 | (*a*) Describe all statistical methods, including those used to control for confounding | Pages 11,12  Section “Statistical analysis” |
|  |  | (*b*) Describe any methods used to examine subgroups and interactions | Pages 11,12  Section “Statistical analysis” |
|  |  | (*c*) Explain how missing data were addressed | N/A |
|  |  | (*d*) If applicable, explain how loss to follow-up was addressed | N/A |
|  |  | (*e*) Describe any sensitivity analyses | N/A |
| Results | | |  |
| Participants | 13* | (a) Report numbers of individuals at each stage of study—eg numbers potentially eligible, examined for eligibility, confirmed eligible, included in the study, completing follow-up, and analysed | Pages 12, 14, 16-18  Addressed in Results part  Also described in pages 8,9: Section “Study design”, table 3 |
|  |  | (b) Give reasons for non-participation at each stage | N/A |
|  |  | (c) Consider use of a flow diagram | Page 6  Fig 1, Timeline |
| Descriptive data | 14* | (a) Give characteristics of study participants (eg demographic, clinical, social) and information on exposures and potential confounders | Pages 6-8  Methods, section “Study area and target population” |
|  |  | (b) Indicate number of participants with missing data for each variable of interest | N/A |
|  |  | (c) Summarise follow-up time (eg, average and total amount) | Page 6  Methods, section “Study area and target population” |
| Outcome data | 15* | Report numbers of outcome events or summary measures over time | Pages 17,18  Results, sections 5 and 6 |
| Main results | 16 | (*a*) Give unadjusted estimates and, if applicable, confounder-adjusted estimates and their precision (eg, 95% confidence interval). Make clear which confounders were adjusted for and why they were included | Pages 12-18  Described throughout the results part |
|  |  | (*b*) Report category boundaries when continuous variables were categorized | N/A |
|  |  | (*c*) If relevant, consider translating estimates of relative risk into absolute risk for a meaningful time period | N/A |
| Other analyses | 17 | Report other analyses done—eg analyses of subgroups and interactions, and sensitivity analyses | Pages 12-18  Analyses of subgroups were described in the results section |
| Discussion | | |  |
| Key results | 18 | Summarise key results with reference to study objectives | Pages 19-24  Addressed in Discussion part |
| Limitations | 19 | Discuss limitations of the study, taking into account sources of potential bias or imprecision. Discuss both direction and magnitude of any potential bias | Mentioned in page 24, line 590 |
| Interpretation | 20 | Give a cautious overall interpretation of results considering objectives, limitations, multiplicity of analyses, results from similar studies, and other relevant evidence | Pages 19-24  Addressed in Discussion part |
| Generalisability | 21 | Discuss the generalisability (external validity) of the study results | N/A |
| Other information | | |  |
| Funding | 22 | Give the source of funding and the role of the funders for the present study and, if applicable, for the original study on which the present article is based | Sources of funding were declared during Plos online submission system |

*Give information separately for exposed and unexposed groups.

**Note:** An Explanation and Elaboration article discusses each checklist item and gives methodological background and published examples of transparent reporting. The STROBE checklist is best used in conjunction with this article (freely available on the Web sites of PLoS Medicine at http://www.plosmedicine.org/, Annals of Internal Medicine at http://www.annals.org/, and Epidemiology at http://www.epidem.com/). Information on the STROBE Initiative is available at http://www.strobe-statement.org.
