# Supplementary material for: Human cellular and humoral immune responses to Phlebotomus papatasi salivary gland antigens in endemic areas differing in prevalence of Leishmania major infection
Source: PLoS Negl Trop Dis. 2017 Oct 12;11(10):e0005905. doi: 10.1371/journal.pntd.0005905 (PMC5638224; doi:10.1371/journal.pntd.0005905)
Supplement: S1 Table — (DOCX) [file pntd.0005905.s002.docx]

| **Foci** | **Gender** | **Age** | **Scars** | **LST** | **LST2** | **Lesion** | **ROD** | **IS-SGE** | **IS-SGE** | **IFN-γ-NS*** | **IFN-γ-SGE** | **IFN-γ-SGE** | **IL10-NS*** | **IL10-SGE** |
| --- | --- | --- | --- | --- | --- | --- | --- | --- | --- | --- | --- | --- | --- | --- |
|  |  |  |  |  |  | **Number** |  |  | **anti-IL10** |  |  | **anti-IL10** |  |  |
| OF | M | 9,5 | - | + | + | 1 | 6,9 | ND | ND | ND | ND | ND | ND | ND |
| OF | M | 8,5 | + | + | + | 1 | 4,3 | 2,9 | 1,4 | 2 | 149 | 149 | 50 | 33 |
| EF | F | 9,1 | - | + | + | 5 | 1,9 | ND | ND | ND | ND | ND | ND | ND |
| EF | M | 8,5 | - | + | + | 1 | 2,6 | ND | ND | ND | ND | ND | ND | ND |
| EF | M | 10,1 | - | + | + | 1 | 4,2 | ND | ND | ND | ND | ND | ND | ND |
| EF | M | 7,4 | - | + | + | 3 | 5,8 | ND | ND | ND | ND | ND | ND | ND |
| EF | M | 10,9 | - | + | + | 1 | 2,7 | 2,4 | 3,3 | 13 | 7 | 9 | 927 | 45 |
| EF | F | 9,9 | + | + | + | 3 | 0,8 | 1,7 | 2,5 | 0 | 3 | 0 | 503 | 12 |
| EF | M | 10,9 | - | + | + | 9 | 2,9 | 1,4 | 2,2 | 7 | 118 | 361 | 101 | 65 |
| EF | M | 10,5 | + | + | + | 2 | 2,0 | 1,3 | 1,4 | 23 | 4 | 12 | 0 | 0 |
| EF | F | 12,6 | - | + | + | 4 | 1,7 | 1,1 | 0,8 | 350 | 207 | 276 | 35 | 4 |
| EF | F | 9,3 | - | + | - | 2 | 5,7 | ND | ND | ND | ND | ND | ND | ND |
| EF | M | 16,3 | - | - | - | 3 | 2,1 | ND | ND | ND | ND | ND | ND | ND |
| EF | F | 12,5 | - | - | - | 1 | 2,5 | ND | ND | ND | ND | ND | ND | ND |
| EF | F | 12,6 | - | - | - | 3 | 2,8 | ND | ND | ND | ND | ND | ND | ND |
| EF | F | 11,5 | - | - | - | 5 | 1,2 | ND | ND | ND | ND | ND | ND | ND |
| EF | F | 8,9 | - | - | - | 1 | 1,8 | 2,0 | 1,3 | 9 | 15 | 16 | 31 | 31 |
| EF | M | 10,4 | - | - | - | 2 | 3,8 | 1,7 | 4,2 | 4 | 1 | 27 | 17 | 23 |
| EF | F | 7,9 | + | - | - | 1 | 1,2 | 0,7 | 1,1 | 11 | 23 | 18 | 41 | 41 |
| EF | F | 8,2 | - | - | - | 1 | 4,4 | 0,7 | 0,4 | 0 | 0 | 0 | 0 | 0 |
| EF | F | 16,5 | - | + | ND | 9 | 1,3 | 1,4 | 0,6 | 21 | 1 | 12 | 9 | 0 |
| EF | M | 13,2 | - | + | ND | 10 | 1,6 | 0,9 | 1,5 | 10 | 36 | 20 | 8 | 38 |
| EF | M | 8,0 | - | - | ND | 2 | 4,1 | ND | ND | ND | ND | ND | ND | ND |
| EF | F | 14,4 | - | - | ND | 8 | 4,0 | 0,8 | 0,6 | 22 | 12 | 9 | 0 | 4 |
| EF | M | 12,9 | - | + | + | 4 | 3,2 | 2,3 | 2,1 | 0 | 0 | 0 | 8 | 18 |
| EF | M | 18,6 | + | + | + | 1 | 1,3 | 0,8 | 1,0 | 0 | 0 | 205 | 20 | 18 |
| EF | F | 10,5 | - | - | + | 1 | 1,9 | 0,8 | 0,2 | 8 | 533 | 52 | 9 | 4 |
| EF | F | 16,6 | - | - | - | 1 | 2,5 | ND | ND | ND | ND | ND | ND | ND |
| EF | F | 14,3 | - | - | - | 1 | 5,8 | 1,8 | 2,4 | 0 | 0 | 0 | 24 | 24 |

**S1 Table. Epidemiological, clinical and immunological parameters for ZCL cases**

*NS: Non-stimulated culture
